# Comparison of two-stage open versus percutaneous pedicle screw fixation in treating pyogenic spondylodiscitis

**DOI:** 10.1186/1471-2474-15-443

**Published:** 2014-12-18

**Authors:** Tung-Yi Lin, Tsung-Ting Tsai, Meng-Ling Lu, Chi-Chien Niu, Ming-Kai Hsieh, Tsai-Sheng Fu, Po-Liang Lai, Lih-Huei Chen, Wen-Jer Chen

**Affiliations:** Department of Orthopaedic Surgery, Spine Section, Chang Gung Memorial Hospital and College of Medicine, Chang Gung University, No. 5, Fusing Street, Guishan Township, Taoyuan 333 Taiwan

**Keywords:** Minimally invasive surgery, Percutaneous pedicle screw, Pyogenic spondylodiscitis, Anterior interbody fusion

## Abstract

**Background:**

Percutaneous pedicle screw instrumentation is a minimally invasive surgical technique; however, the effects of using percutaneous pedicle screw fixation in treating patients with spinal infections have not yet been well demonstrated. The aim of this study, therefore, was to determine whether percutaneous posterior pedicle screw instrumentation is superior to the traditional open approach in treating pyogenic spondylodiscitis.

**Methods:**

We retrospectively reviewed data for 45 patients treated for pyogenic spondylodiscitis with anterior debridement and interbody fusion followed by a second-stage procedure involving either traditional open posterior pedicle screw fixation or percutaneous posterior pedicle screw fixation. Twenty patients underwent percutaneous fixation and 25 patients underwent open fixation. Demographic, operative, and perioperative data were collected and analyzed.

**Results:**

The average operative time for the percutaneous procedure was 102.5 minutes, while the average time for the open procedure was 129 minutes. The average blood loss for the percutaneous patients was 89 ml versus a 344.8 ml average for the patients in the open group. Patients who underwent the minimally invasive surgery had lower visual analogue scale scores and required significantly less analgesia afterwards. After two years of follow-up, neither recurrent infection nor intraoperative complications, such as wound infection or screw loosening, were found in the percutaneous group. Moreover, there was no significant difference in outcome between the two groups in terms of Oswestry Disability Index scores.

**Conclusions:**

Anterior debridement and interbody fusion with bone grafting followed by minimally invasive percutaneous posterior instrumentation is an alternative treatment for pyogenic spondylodiscitis which can result in less intraoperative blood loss, shorter operative time, and reduced postoperative pain with no adverse effect on infection control.

**Electronic supplementary material:**

The online version of this article (doi:10.1186/1471-2474-15-443) contains supplementary material, which is available to authorized users.

## Background

Pyogenic spondylodiscitis is a challenging medical disease with poor prognosis that requires immediate diagnosis and treatment with suitable antibiotics [1]. In recent years, an increased incidence of pyogenic spinal infections has been associated with immunocompromised status, advanced age, invasive medical procedures, and underlying medical comorbidities [2, 3]. Surgical intervention is indicated when non-surgical treatment fails, neurologic deficit develops, or tissue biopsy is required.

Anterior debridement and interbody fusion with bone graft have been reported to serve as an effective treatment for pyogenic spondylodiscitis [1, 4, 5], followed by a one- or two-stage posterior instrumentation in the event of posterior element deformity or spinal instability [6]. This additional procedure can produce better stabilization and fusion results with no adverse effect on infection control. However, it may lead to higher perioperative complications and significant comorbidities as it can involve extensive soft tissue dissection and posterior element destruction.

Percutaneous pedicle screw instrumentation is a minimally invasive surgical technique which has been used worldwide to achieve less damage and faster recovery [7]. Some studies have shown that patients with a spinal infection may be good candidates for percutaneous pedicle screw and rod fixation [8, 9]; however, still other studies have reported some limitations to this approach [10, 11]. The purpose of this study, therefore, was to determine whether percutaneous posterior pedicle screw instrumentation is superior to the traditional open approach in treating pyogenic spondylodiscitis.

## Methods

After obtaining approval from the Institutional Review Board (IRB) of Chang Gung Medical Foundation, we reviewed the medical charts of patients at our institution who were treated for pyogenic spondylodiscitis with surgical management between January 2007 and June 2011. The indications for surgical treatment included antibiotic treatment failure, presence of abscess, and persistent intractable back pain. Initially, 66 patients underwent anterior debridement and interbody fusion with bone graft for infection control. Postoperatively, they received antibiotics and aggressive rehabilitation. At one week after anterior interbody fusion, if a patient still had persistent back pain and/or exhibited focal kyphosis on plain radiographs, then additional posterior instrumentation was indicated for spinal stabilization and early mobilization.

All enrolled patients were diagnosed with pyogenic spondylodiscitis and received anterior interbody fusion followed by a second-stage posterior procedure involving either open or percutaneous pedicle screw fixation, according to their own decision after receiving sufficient information regarding both approaches. All operations were performed by spine surgeons at our institution using a similar anterior procedure but two different posterior approaches, as described below. All the enrolled patients had signed informed consent and agreed to join this study.

### Surgical technique

#### Anterolateral interbody fusion and debridement

After general anesthesia was administered, the patient was placed in the lateral decubitus position. The anterolateral retroperitoneal approach provided access to the lumbar spine, and the transthoracic approach was used for the thoracic spine. After achieving good exposure of the infected disc, it was adequately debrided and a tricortical autologous iliac crest bone graft was placed into the involved intervertebral space.

#### Percutaneous posterior pedicle screw fixation

**Figure 1:**
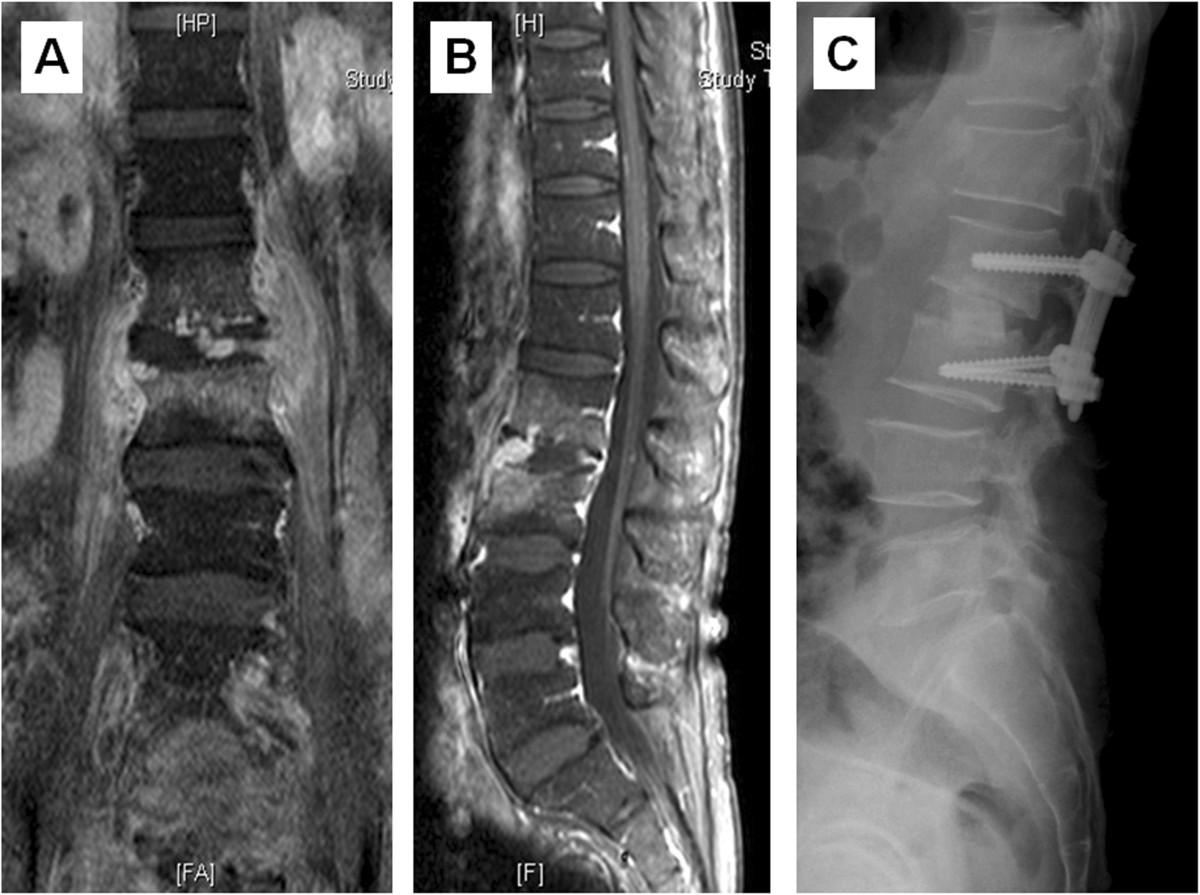
**A case of hematogenous pyogenic spondylodiscitis. (** **A)** Coronal plane of lumbar spine MRI and **(B)** sagittal view revealed infectious spondylodiscitis at L2-3. **(C)** Postoperative lateral radiograph demonstrated the presence of cortical allograft and percutaneous posterior instrumentation.

**Figure 2:**
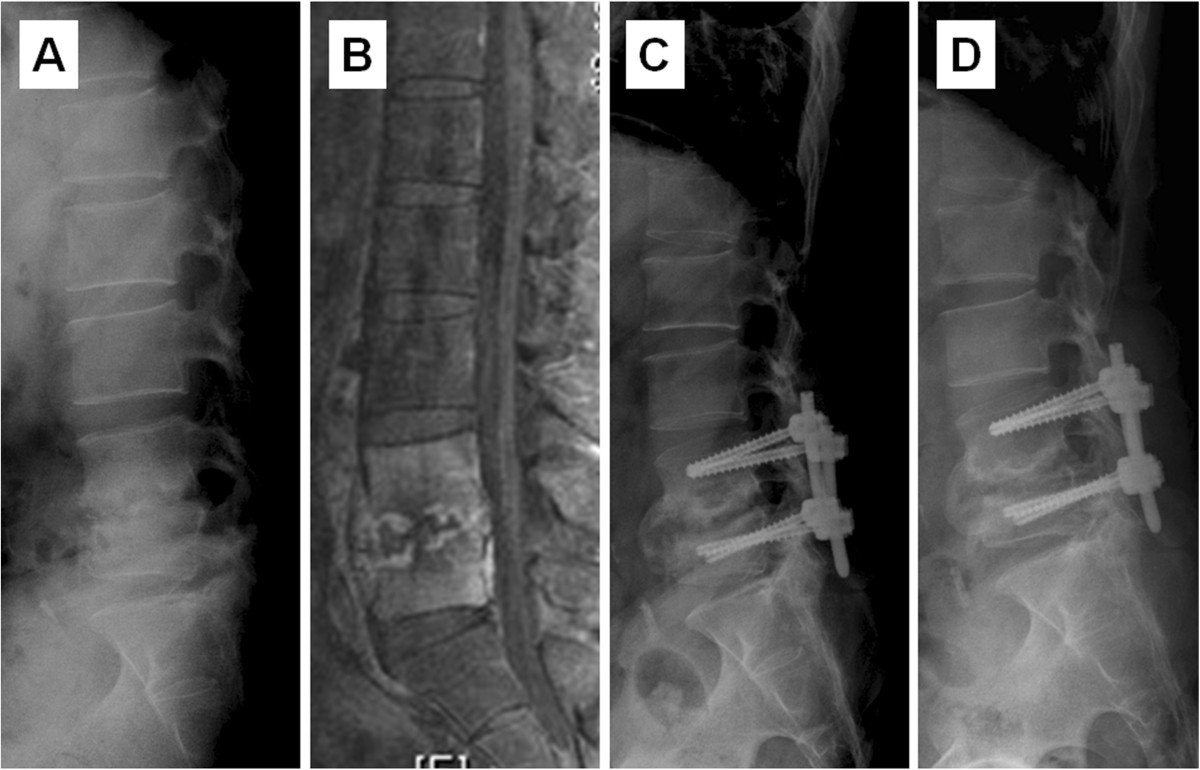
**A case of hematogenous pyogenic spinal infection** **. (A)** Preoperative lateral radiograph showed obvious disc space narrowing with endplate erosion at L4-5, and focal kyphosis **(B)** MRI revealed L4-5 infectious spondylodiscitis. **(C)** Postoperative lateral radiograph demonstrated the presence of anterior interbody fusion with allograft and percutaneous posterior pedicle screw. **(D)** Postoperative lateral view at two-year follow-up revealed bone union without progression in focal kyphosis.

The patient was placed in the prone position after general anesthesia was administered. One of two kinds of minimally invasive surgery (MIS) system, either the Sextant system (Medtronic Sofamor Danek) or the Viper system (Depuy Spine), was used for each percutaneously treated patient in the study. If endplate erosion and vertebral bone destruction were relatively subtle, the involved vertebral levels were instrumented. However, if vertebral bone destruction was severe, screws were inserted at one level above and one level below the involved vertebral levels.Intraoperative fluoroscopy was used to localize the appropriate spinal levels to ensure proper placement of the pedicle screws. Four paraspinal skin incisions, each approximately 1.5 cm in length, were made. Under C-arm guidance, the Jamshidi needle was gradually advanced through the pedicle at the optimal entry point, and guide wires were inserted. While maintaining the position of the wires within the pedicle, the needle was removed and the pedicle preparation cannula was placed after dilatation. The pedicle screws were placed in the standard fashion, and the rods were placed with the aid of a rod guider. The same procedure was repeated on the other side of the spine. Plain radiographs were taken immediately after insertion to ensure the accuracy of pedicle screw placement (Figures 1 and 2).

#### Traditional open posterior pedicle screw fixation

**Figure 3:**
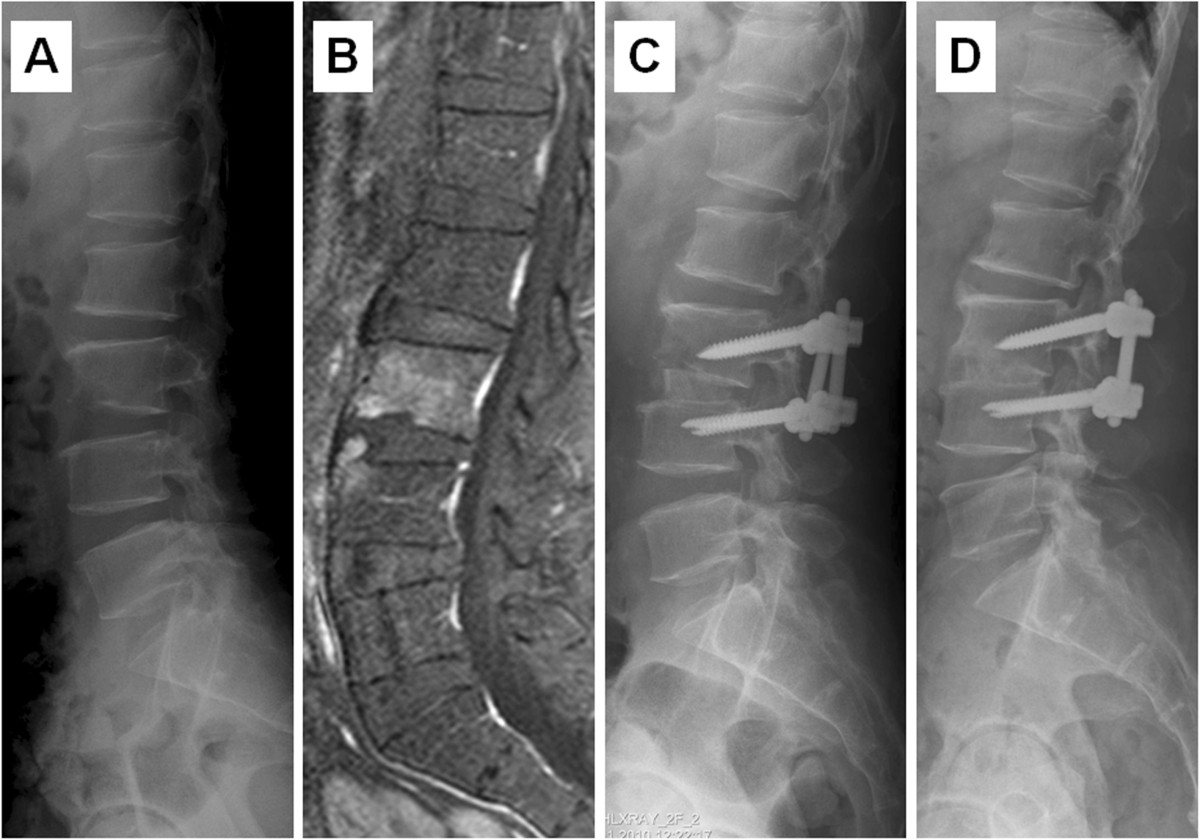
**A case of hematogenous pyogenic spinal infections. (** **A)** Preoperative lateral radiograph showed disc space narrowing with endplate erosion at L3-4 level, and **(B)** MRI revealed L3-4 spondylodiscitis. **(C)** Postoperative lateral radiograph demonstrated the presence of anterior interbody fusion with allograft and traditional open posterior pedicle screw. **(D)** Postoperative lateral view at two-year follow-up revealed union of L3-4 interbody fusion.

A standard posterior midline incision was made through the thoracolumbar fascia, and the paraspinous muscles were stripped bilaterally accompanied by hemostasis. After detection of the entry point, the pedicle screws were inserted, and the rods were placed (Figure 3). Similarly, the involved vertebral levels were instrumented one level above and one level below the involved vertebral levels if severe bone destruction was observed involving the infected vertebrae.

### Clinical variables and evaluation

Demographic, operative, and perioperative data were collected and analyzed, including infected vertebral level, posterior instrumented level, screw system used, comorbidity, bone graft, operative time, VAS (visual analogue scale) score on postoperative day 1, number of analgesic injections, infectious organism encountered, days of antibiotic treatment, and duration of follow-up. Intraoperative blood loss was estimated by suction bottle measurement and the total weight of the gauze used during surgery. Intraoperative specimens were sent for organism identification and antibiotics sensitivity testing. For postoperative pain relief, oral administration of acetaminophen at 500 mg QID and celebrex at 200 mg QD were routinely used. In addition, 50 mg of Demerol (meperidine hydrochloride) was injected intramuscularly, and the number of injections after the second-stage posterior instrumentation was recorded.

Postoperative bony union was defined as intervertebral bony bridges observed on follow-up radiographs taken post-operatively and at 3 months, 9 months, one year, and two years postoperatively. Antibiotics were administered for at least 4 weeks and could then be stopped if the WBC count and CRP level improved to within the normal limits. MRI scans were taken as necessary whenever the condition progressed, such as when a fever flared up or if lab data indicated that the condition was worsening.

Functional outcomes were retrospectively evaluated by independent reviewers via administration of the Oswestry Disability Index (ODI) preoperatively and after two years of follow-up.

### Statistical analysis

Either Pearson chi-square test or Fisher’s exact test were used for group comparison of categorical variables. Either two-tailed t-test or Mann–Whitney U test was used for group comparison of numerical variables. Numerical data were presented as mean ± standard deviation, while categorical data were expressed in absolute frequencies by SPSS. A *p* < 0.05 was considered statistically significant, as indicated by an asterisk in the table.

## Results

A total of 45 patients who needed staged posterior instrumentations after receiving anterior interbody fusion were enrolled in this study. Twenty patients underwent the percutaneous method (the percutaneous group), and 27 patients underwent the traditional open method (the open group). Two patients were lost to follow-up and then excluded. The percutaneous group was comprised of fourteen males and six females with a mean age of 59.6 years. In the open group, there were eleven males and fourteen females with a mean age of 64.7 years (Table 1).

**Table 1:** **Comparison of data between the percutaneous and open groups**

	Percutaneous group (n = 20)	Open group (n = 25)	***P*** value
Age (yr)	59.6	64.7	0.113
Sex	14 males	11 males	0.085
	6 females	14 females	
Anterior operative time (min)	149 ± 39.5	156 ± 33.7	0.534
Anterior blood loss (mL)	577.5 ± 203.4	594 ± 194.1	0.788
Posterior operative time (min)	102.5 ± 28.3	129 ± 20.9	0.001*
Posterior blood loss (mL)	89 ± 34.6	344.8 ± 155.2	<0.001*
Number of analgesic injections	3.9 ± 2.1	5 ± 1.4	0.042*
VAS score, next day after posterior instrumentation	4 ± 1.3	5.5 ± 1.2	<0.001*
VAS score, 7 days after posterior instrumentation	2.8 ± 1.2	3.5 ± 0.9	0.03*

VAS, visual analogue scale.

Data presented as mean ± standard deviation, and *P < 0.05.

Within the percutaneous group, two patients had a spinal infection within the thoracic region whereas the rest of the patients in the group had lumbar infections. In contrast, there were seven patients in the open group who had involved thoracic spinal infections and the remainder had infections within the lumbar spine. Our positive culture rate from deep tissue biopsy during anterior fusion surgery was above 90% (18/20 for the percutaneous group and 23/25 for the open group, Table 2), and the most common organism encountered was staphylococcus aureus in both groups. Moreover, 36 (80%) patients had some degree of underlying medical comorbidity, including diabetes mellitus in nine cases in the percutaneous group and in fifteen cases in the open group (Table 3).

**Table 2:** **Causative organisms**

Organisms	Percutaneous group (n = 20)	Open group (n = 25)
MSSA	5(25%)	6 (24%)
MRSA	3(15%)	4 (16%)
Staph.epidermidis	2(10%)	2 (8%)
E. coli	0	3 (12%)
Pseudo.aeruginosa	2(10%)	1 (4%)
Enterococcus faecalis	0	1 (4%)
Kleb. Pneumoniae	4(20%)	2 (8%)
Penicillium species	0	2 (8%)
Streptococcus Group B	1(5%)	0
Culture negative	3(15%)	4 (16%)

MRSA = Methicillin-resistant Staphylococcus aureus, MSSA = Methicillin-sensitive Staphylococcus aureus.

**Table 3:** **Patient comorbidity and perioperative complications**

	Percutaneous group (n = 20)	Open group (n = 25)
Patient comorbidity		
Hypertension	7(35%)	12(48%)
Diabetes mellitus	9(45%)	13(52%)
Liver cirrhosis	2(10%)	2(8%)
End stage renal disease	3(15%)	3(12%)
Cancer history	2(10%)	2(8%)
COPD	1(5%)	1(4%)
Perioperative complications		
Incidental durotomy	0	0
Wound problem	1	2
Donor site infection	0	0
Pneumonia	0	0
Urinary tract infection	0	0
Screw malposition	0	2

COPD = Chronic obstructive pulmonary disease.

The demographic and perioperative data for the two groups are summarized in Table 1. For the anterior procedure, the average operative time for the percutaneous group was 149 minutes (range, 90–240 min), while the average for the open group was 156 minutes (range, 110–225 min). The average blood loss was 577.5 ml (range, 200–900 ml) for the percutaneous group vs. 594 ml (range, 250–950 ml) for the open group.

For the posterior procedure, the average operative time was 102.5 minutes (range, 60–160 min) in the percutaneous group and 129 minutes (range, 100–165 min) in the open group (*P* = 0.001). The average blood loss was 89 ml (range, 50–200 ml) in the percutaneous group and 344.8 ml (range, 100–600 ml) in the open group (*P* < 0.001). Patients required significantly fewer analgesic injections after posterior minimally invasive surgery compared to open surgery (3.9 vs. 5, *P* = 0.042). Also, the VAS score on postoperative day 1 and day 7 indicated that patients in the percutaneous group had significantly less pain (day 1 : 4 vs. 5.5, *P* <0.001; day 7 : 2.8 vs. 3.5, *P* = 0.03). No complication related to immobilization such as pneumonia, urinary tract infection or muscle wasting was found in the percutaneous group. In addition, no donor site infection was observed in this study. The functional outcomes measured via telephone interview after two years of follow-up showed that the percutaneous group had better ODI scores compared to the open group, however, the difference in scores was not significant (19.7 vs. 21.8, *P* = 0.122).

Even though patients were treated with surgical intervention, antibiotics still played an important role in treating pyogenic spondylodiscitis. All patients received a minimum of four weeks of antibiotic administration (range, 28–83 days, including oral and parenteral antiobiotics). The treatment plan was discussed with our Department of Infection and adjusted according to culture results, lab data, and clinical symptoms and signs. If symptoms improved and C-reactive protein levels returned to normal during hospitalization, patients were allowed to switch to oral antibiotics and were discharged from the hospital.

After a two-year follow-up, neither recurrent infection nor intraoperative complications, such as wound infection or screw loosening, were found in the percutaneous group. In the percutaneous group, a 68-year-old male patient who had diabetes mellitus for ten years experienced poor healing at the anterior wound site. The wound gradually healed 3 weeks after surgery after placement of additional 3–0 nylon sutures. In the open group, two patients had stitches abscess which required prolonged wound care. Another one patient in open group was found to have asymptomatic screw loosening according to the X-ray, during the outpatient period. At two-year follow-up, all instrumented vertebral segments in the ten percutaneous patients showed good bony union on radiographic images. On the other hand, three patients (one in the percutaneous group and two in the open group) had recurrent fever with elevated white blood cell count and C-reactive protein level during the follow-up and had treated with an additional 4-week course of antibiotics.

## Discussion

Pyogenic spondylodiscitis can be treated nonsurgically with antibiotics and immobilization. Surgical intervention is indicated when neurologic deficit, epidural abscess or kyphotic deformity has developed [12, 13]. Anterior debridement and fusion has been proven effective in treating pyogenic spondylodiscitis. This anterior procedure allows direct access to the infected disc and enables sufficient debridement and placement of bone graft for adequate stabilization. Additionally, it allows the performance of tissue biopsy to ensure reliable microbiological diagnosis and rapid relief of symptoms [5, 14]. Occasionally, however, the anterior approach alone is not sufficient to restore spinal stability and correct the kyphotic deformity, and an additional posterior instrumentation procedure is indicated.

The goals of surgical treatment are decompression of the spine, eradication of infection, relief of intractable pain, correction of deformity, and biopsy of infected tissue. Sundararaj et al. [15] have reported good results for 32 patients who underwent single-stage anterior debridement, fusion with bone graft and posterior instrumentation, all achieved in one operation. However, their technique was associated with relatively increased risk of complications, including delayed wound healing, superficial and deep wound infections, and pneumonia, and consequently further surgery or long hospitalization was required. Similar issues were reported in studies conducted by Korovessis et al. [16, 17]. In spite of the fact that patients may suffer from severe wound pain and muscle damage after traditional posterior instrumentation [18, 19], anterior interbody fusion with grafting followed by posterior instrumentation plays a significant role in the treatment of pyogenic spondylodiscitis, and its advantages outweigh the perceived risks [20, 21].

Percutaneous pedicle screw fixation has been widely used for spondylolisthesis, trauma, and tumor [22, 23]. This less invasive technique has been shown to be of potential benefit to patients with multiple comorbidities [11]. In our study, the use of minimally invasive posterior instrumentation achieved good outcomes at 2-year follow-up without adverse effect on infection control in patients who had undergone anterior debridement and fusion. Patients could ambulate as soon as wound pain became tolerable after undergoing the posterior procedure for spinal stability. In our study population, there were no recurrent infections, no complications related to immobilization, and no implant failure after 2 years of follow-up.

The intraoperative fluoroscopic guidance may explain the optimal positioning of pedicle screws and the reduced risk of screw malposition. Compared to traditional open instrumentation, percutaneous instrumentation may also result in less blood loss, reduced operative time, less pain on postoperative day one and less opioid consumption. Although there was no significant difference between our two groups, the long-term functional outcomes suggested posterior percutaneous fixation as an alternative treatment.

Previous studies have discussed the use of minimally invasive surgery to treat pyogenic spondylodiscitis. Yang et al. [24] used percutaneous endoscopy for debridement and drainage, and the results showed a high positive culture rate of 90% which represented an advantage over CT-guided biopsy. Hadjipavlous et al. [25] demonstrated that percutaneous transpedicular discectomy and drainage could result in immediate pain relief. Nevertheless, without instrumented fixation, a drainage and debridement procedure alone cannot correct spinal deformity and instability. Deininger et al. [9] reported their experience with percutaneous dorsal instrumentation but no anterior debridement and fusion in 12 patients with pyogenic spondylodiscitis. The combined effect of antibiotics and percutaneous fixation achieved quick pain relief and rapid mobilization. However, patients receiving posterior instrumentation without interbody fusion can still incur anterior bony defects and may require additional long-segment instrumentation. In addition, lack of anterior interbody fusion may also result in a low rate of successful bacterial culture (only 58%) because the amount of infected tissue sampled for the biopsy may not be sufficient. Nasto et al. [8] presented a retrospective cohort study to compare percutaneous posterior fixation to conservative bracing in treating patients with pyogenic spondylodiscitis due to a known infectious agent. The results showed lower VAS scores in the percutaneous screw group at 1-month and 3-month follow-ups and no difference in terms of infection control. In our study, posterior percutaneous fixation resulted in similar benefits in terms of pain relief; however, the anterolateral interbody fusion and debridement approach results in the obtainment of a positive culture in more than 90% of cases.

In a related study, Kandwai et al. [26] presented their MIS experience in the treatment of tuberculosis spondylitis. They achieved high fusion rates and good functional results in patients who underwent percutaneous screw fixation and posterolateral debridement and fusion through the mini-open approach. In our series, the high positive culture rate (up to 90% in the percutaneous group and 92% in the open group) was achieved due to the open anterior procedure. It allowed direct access to the infected area for debridement and the collection of pus for culture and antibiotic testing, with a resultant high success rate in treating pyogenic spondylodiscitis.

Our study had several limitations. Our relative small sample size limited the number of outcomes available for comparison. In addition, the approach used in the second-staged posterior fixation procedure, whether open or percutaneous, depended upon eahc patient's individual preference which may have contributed some bias. However, this study provides important information regarding staged percutaneous versus open fixation in patients with spondylodiscitis who need surgical intervention. Moreover, minimally invasive percutaneous fixation is suggested in patients with multiple comorbidities who are at high risk of perioperative complications [27, 28], as it promotes faster recovery and possibly diminishes complications. Finally, a preoperative survey and consultation with an anesthesiologist before the surgery is also suggested to improve surgical outcome and minimize risks and complications.

## Conclusion

Anterior interbody fusion with bone graft followed by a second-stage minimally invasive percutaneous posterior instrumentation provided an alternative method for treating pyogenic spondylodiscitis which resulted in less intraoperative blood loss, shorter operative time, reduced postoperative pain, and less opioid consumption compared to the traditional open posterior instrumentation approach. In our series, the percutaneous technique achieved satisfactory results in a long-term follow-up and showed no adverse effect on infection control.

### Ethic statement

Protocol No: Institutional Review Board CGMF IRB No.: 100–4083 from The name of the ethics committee: Chang Gung Medical Foundation 199, TUNG HWA NORTH ROAD, TAIPEI, TAIWAN, 10507, Republic Of China Tel: +886-3-3196200 FAX: +886-3-3196102.

## Authors’ original submitted files for images

Below are the links to the authors’ original submitted files for images. Authors’ original file for figure 1 Authors’ original file for figure 2 Authors’ original file for figure 3
