# Supplementary figures and images for: Comparison of two-stage open versus percutaneous pedicle screw fixation in treating pyogenic spondylodiscitis
Source: BMC Musculoskelet Disord. 2014 Dec 18;15:443. doi: 10.1186/1471-2474-15-443 (PMC4300775; doi:10.1186/1471-2474-15-443)

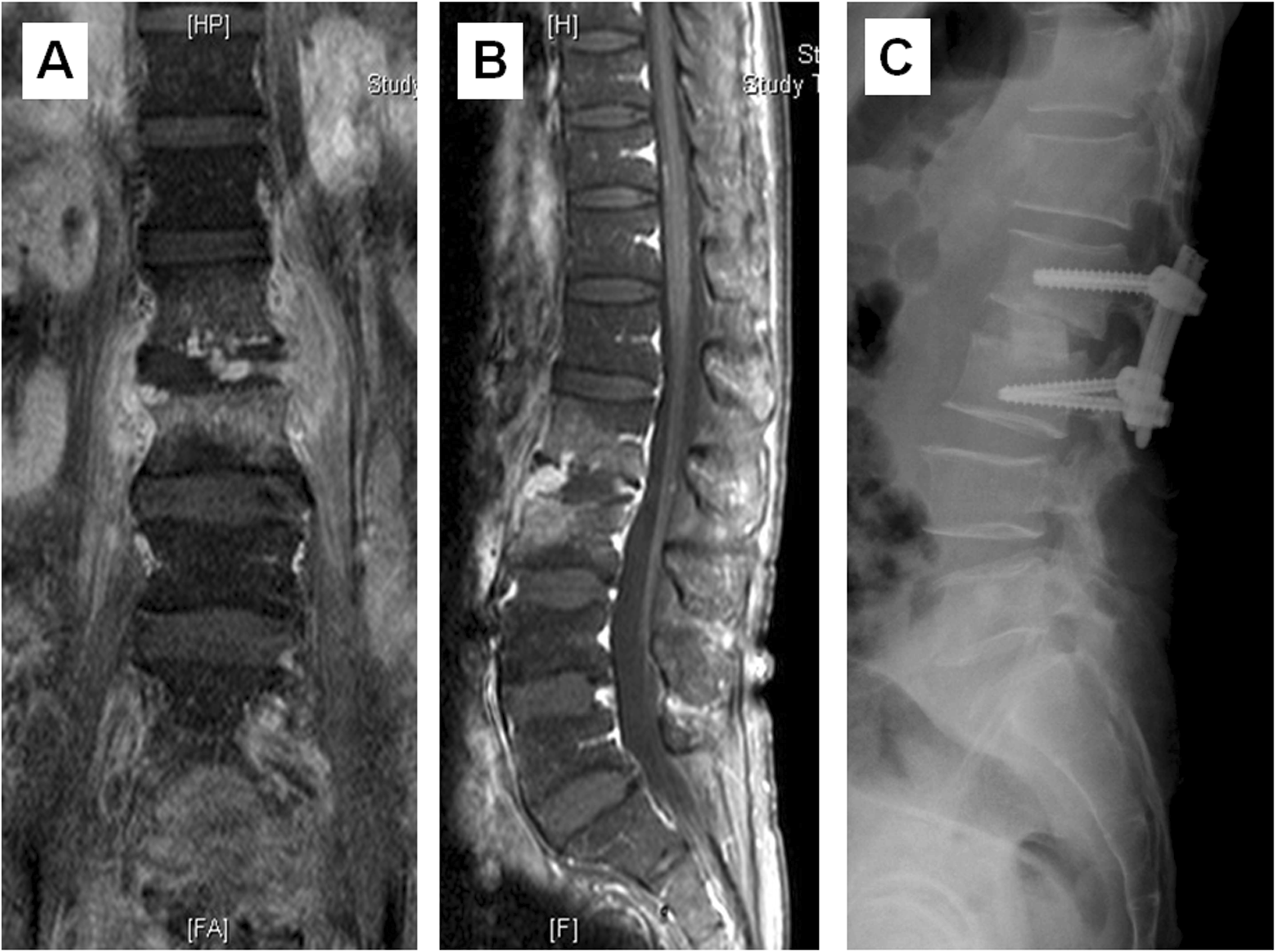

Supplement: Supplementary file 1 — Authors’ original file for figure 1 [file 12891_2014_2381_MOESM1_ESM.tif]

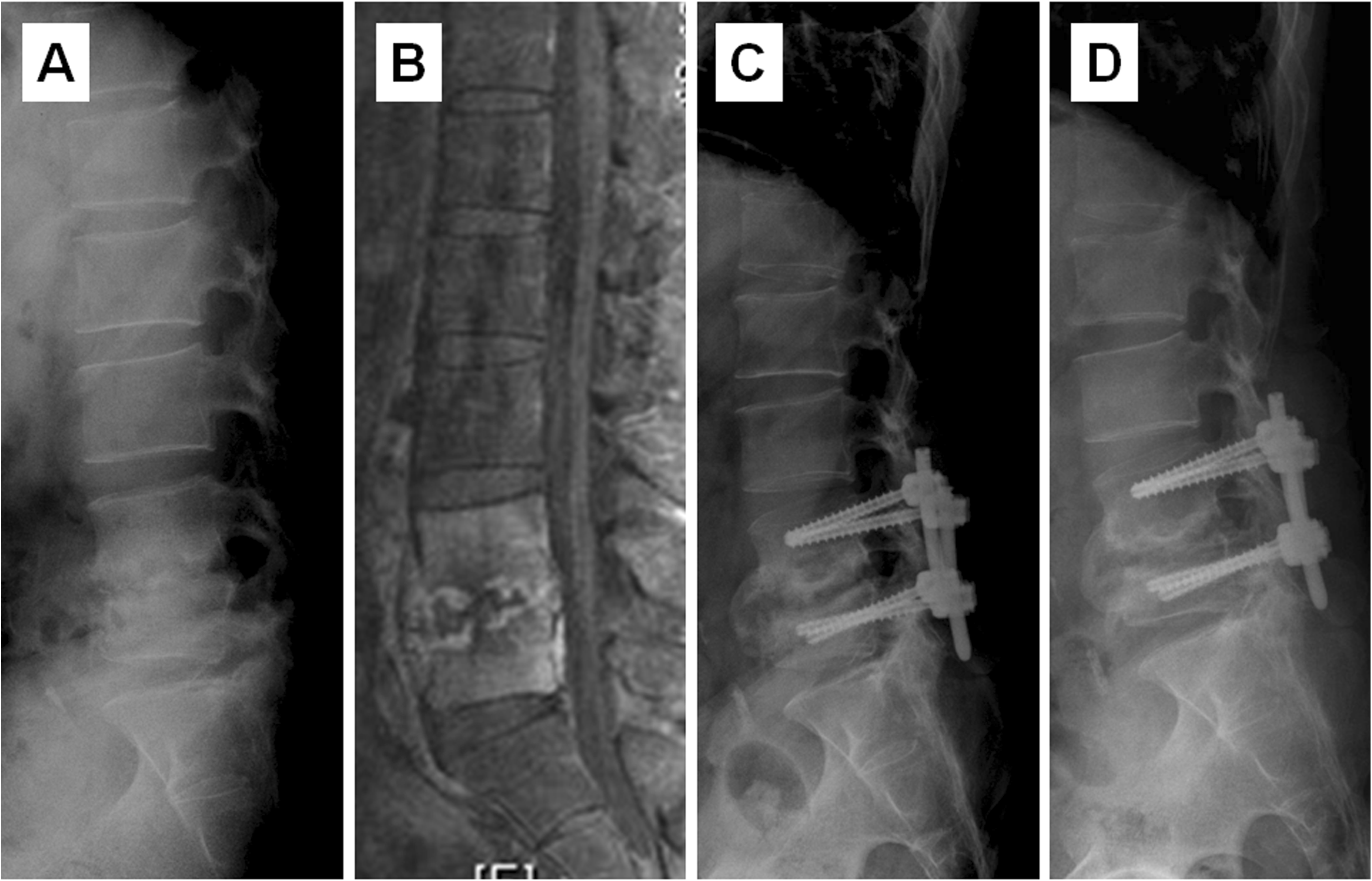

Supplement: Supplementary file 2 — Authors’ original file for figure 2 [file 12891_2014_2381_MOESM2_ESM.tif]

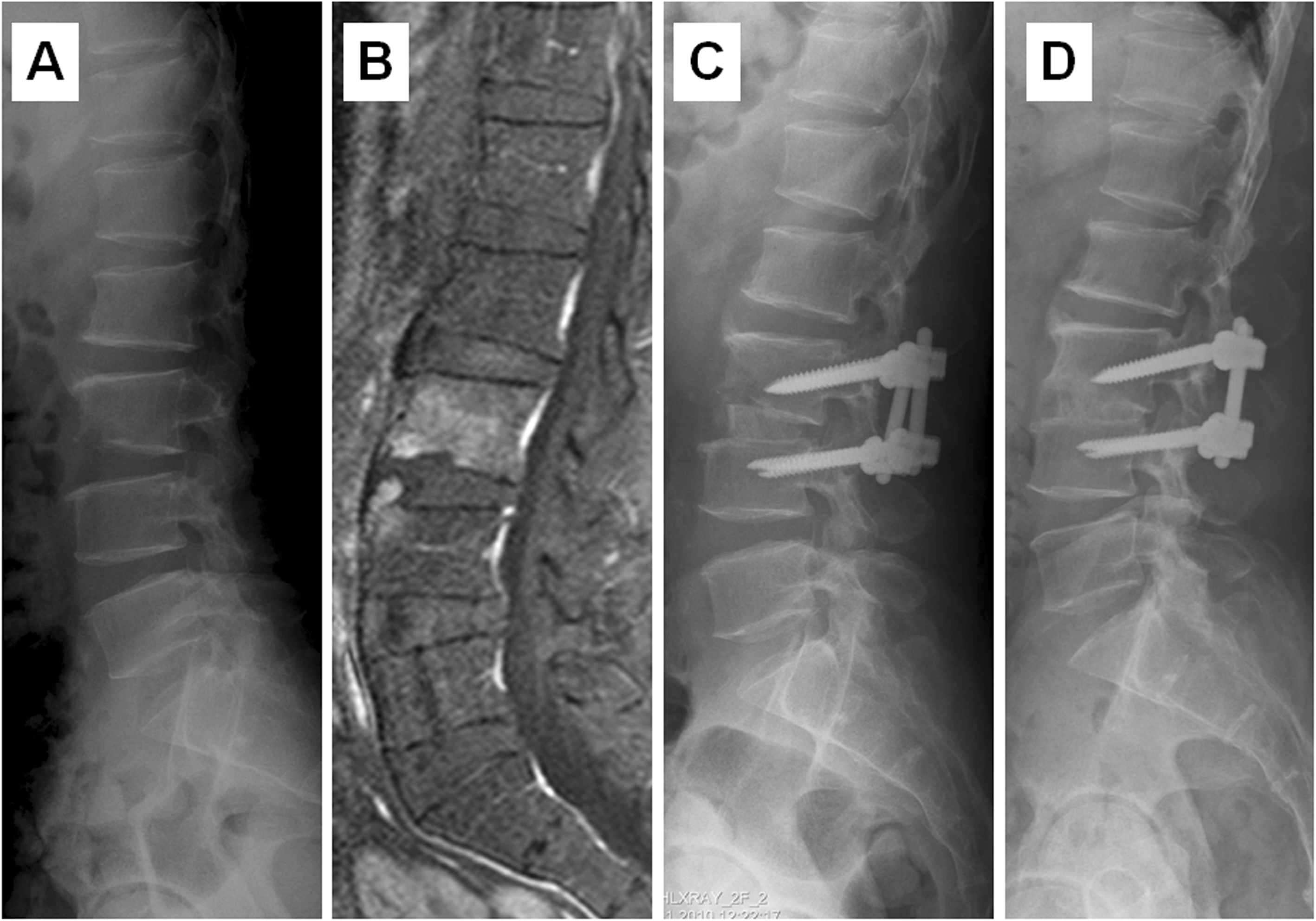

Supplement: Supplementary file 3 — Authors’ original file for figure 3 [file 12891_2014_2381_MOESM3_ESM.tif]
